# Attenuation of orthodontically induced inflammatory root resorption by using low-intensity pulsed ultrasound as a therapeutic modality- a systematic review

**DOI:** 10.1186/s12903-023-03741-1

**Published:** 2024-01-10

**Authors:** Sunil Kumar Vaddamanu, Fahad Hussain Alhamoudi, Rajesh Vyas, Vishwanath Gurumurthy, Yulia Siurkel, Marco Cicciù, Giuseppe Minervini

**Affiliations:** 1 Department of Dental Technology, College of Applied Medical Sciences, King Khalid University, Abha, 62529 Saudi Arabia; 2 International European University School of Medicine, Akademika Hlushkova Ave, 42В, Kyiv, 03187 Ukraine; 3 Department of Biomedical and Surgical and Biomedical Sciences, Catania University, Catania, 95123 Italy; 4 Saveetha Dental College and Hospitals, Saveetha Institute of Medical and Technical Sciences (SIMATS), Saveetha University, Chennai, Tamil Nadu India; 5 Multidisciplinary Department of Medical-Surgical and Dental Specialties, University of Campania Luigi Vanvitelli, Caserta, 81100 Italy

**Keywords:** Root resorption, Ultrasound, Orthodontic, Tooth movement, Low-intensity pulsed ultrasound (LIPUS), Non-invasive therapies

## Abstract

Ultrasound is an effective tool for both diagnostic and therapeutic applications. As an imaging tool, ultrasound has mostly been used for real-time noninvasive diagnostic imaging. As ultrasound propagates through a material, a reflected radio-frequency (RF) signal is generated when encountering a mismatch in acoustic impedance. While traditionally recognized for its diagnostic imaging capabilities, the application of ultrasound has broadened to encompass therapeutic interventions, most notably in the form of Low-Intensity Pulsed Ultrasound (LIPUS). Low-Intensity Pulsed Ultrasound (LIPUS) is a form of mechanical energy transmitted transcutaneously by high-frequency acoustic pressure waves. The intensity of LIPUS (30 mW/cm2) is within the range of ultrasound intensities used for diagnostic purposes (1–50 mW/cm2) and is regarded as non-thermal, non-destructive, permeating living tissues and triggering a cascade of biochemical responses at the cellular level. The LIPUS device produces a 200 µs burst of 1.5 MHz acoustic sine waves, that repeats at a modulation frequency of 1 kHz and provides a peak pressure of 30 mW/cm2. Low-intensity pulsed ultrasound (LIPUS) forms one of the currently available non-invasive healing-enhancing devices besides electro-stimulation (pulsed electro-magnetic field, PEMF). This modality has been leveraged to enhance drug delivery, expedite injury recovery, improve muscle mobility, alleviate joint stiffness and muscle pain, and enhance bone fracture healing. Although LIPUS has been embraced within various medical disciplines, its integration into standard dental practices is still in its nascent stages, signifying an unexplored frontier with potentially transformative implications. Low-intensity pulsed ultrasound (LIPUS) has emerged as an attractive adjuvant therapy in various dental procedures, such as orthodontic treatment and maxillary sinus augmentation. Its appeal lies in its simplicity and non-invasive nature, positioning LIPUS as a promising avenue for clinical innovation. One particular area of interest is orthodontically induced inflammatory root resorption (OIIRR), an oftenunavoidable outcome of the orthodontic intervention, resulting in the permanent loss of root structure. Notably, OIIRR is the second most common form of root resorption (RR), surpassed only by root resorption related to pulpal infection. Given the high prevalence and potential long-term consequences of OIIRR, this literature review seeks to evaluate the efficacy of LIPUS as a therapeutic approach, with an emphasis on assessing its capacity to reduce the severity of OIIRR to a level of clinical significance. To conduct this systematic review, a comprehensive automated literature search was executed across multiple databases, including MEDLINE, Embase, PsycINFO, Web of Knowledge, Scopus, CINAHL, LILACS, SciELO, Cochrane, PubMed, trials registries, 3ie, and Google Scholar. Both forward and backward citation tracking was employed, encompassing studies published from database inception through January 2009 to April 2023. The review focused on randomized controlled trials (RCTs) that specifically evaluated the effects of low-intensity pulsed ultrasound therapy on orthodontically induced inflammatory root resorption (OIIRR), without restrictions of publication date. A stringent selection criterion was applied, and only studies demonstrating high levels of statistical significance were included. Ultimately, fourteen studies met the inclusion criteria and were subjected to further analysis. The overall quality of the included randomized controlled trials (RCTs) was rigorously assessed utilizing the Grading of Recommendations Assessment, Development, and Evaluation (GRADE) approach. This analysis revealed certain methodological limitations that posed challenges in drawing definitive conclusions from the available evidence. Despite these constraints, the review offers invaluable insights that can inform and guide future research. Specifically, it delineates recommendations for targeted populations, necessary interventions, appropriate outcome measures, suitable study designs, and essential infrastructure to facilitate further investigations. The synthesis of these insights aims to enhance the development and application of low-intensity pulsed ultrasound therapy within the field of dentistry, thereby contributing to improved patient outcomes.

## Introduction

Ultrasound, manifesting as acoustic pressure waves with frequencies exceeding the audible range for human hearing, is a distinct mechanical energy capable of transmission into biological tissues as high-frequency acoustic pressure oscillations [1]. The resultant interaction between ultrasound waves and bodily tissues yields effects that vary contingent on the specific intensity deployed [2, 3]. Within the broad spectrum of ultrasound applications, therapeutic modalities generally employ intensities ranging from 30 to 70 W/cm², whereas operative (shock waves) and diagnostic ultrasounds fall within the ranges of 0.05 to 27,000 W/cm² and 5 to 50 mW/cm², respectively [4].

In contemporary biomedical practice, Low-Intensity Pulsed Ultrasound (LIPUS) has emerged as a specialized variant of acoustic pulsed energy, drawing considerable attention for its capacity to stimulate physiological responses within targeted tissues. Distinguished by its unique parameterization, LIPUS operates with a pulsed frequency of 1.5 MHz, a signal burst width of 200 µs, a signal repetition frequency of 1 kHz, and a specific intensity of 30 mW/cm² [4–6].

Despite the burgeoning interest and application of LIPUS in the context of tissue repair, the precise biological mechanisms orchestrating the response to LIPUS stimulation remain to be fully elucidated [4, 7]. As the sound energy traverses living tissues, a differential absorption is observed that is proportionate to the density of the constituent tissue elements [8, 9]. This absorption is not merely a passive event; it culminates in the conversion of sound energy into mechanical oscillations of molecules within the targeted cellular structures.

The intricate nature of this interaction has led to the hypothesis that the anabolic effects invoked by LIPUS may be orchestrated through mechanical stimuli or a process known as acoustic microstreaming [10, 11].

It has been hypothesized that the interaction between LIPUS and cells may occur through integrin molecules, which act as mechanoreceptors situated on the cell membrane [12–15]. These integral membrane proteins could potentially serve as conduits for the transduction of mechanical signals induced by LIPUS into the cellular milieu.

This interaction is postulated to instigate the activation and phosphorylation of focal adhesion kinase (FAK), thereby initiating intricate intracellular signaling cascades [16]. LIPUS has been shown to augment the tyrosine phosphorylation of various signaling proteins, trigger the activation of serine/threonine kinases, and instigate modifications in cellular phospholipids and calcium concentrations [17]. These effects reflect the versatility of LIPUS in modulating complex cellular processes.

Moreover, the role of integrin in activating associated signaling pathways is well documented. For example, the mitogen-activated protein kinase (MAPK) pathway [18], as well as the Rho pathway [19], have been identified as downstream effectors of integrin activation. These pathways are essential in governing an array of cellular functions and contribute to the understanding of the multifaceted biological effects of LIPUS.

Furthermore, integrins may act as a nexus between the extracellular matrix, cytoskeletal proteins, and actin filaments, providing a dynamic interface for the transmission of mechanical cues [20].

In human chondrocytes, LIPUS stimulation has been found to prompt the expression of cyclooxygenase-2 (COX-2), a key enzyme involved in prostaglandin synthesis [20]. This induction is thought to be mediated through a complex signaling cascade encompassing p300, integrin-linked kinase, and integrin signaling. The resulting elevation in prostaglandin production has significant implications for bone metabolism, reflecting the multifaceted role of LIPUS in modulating physiological responses.

Beyond its mechanical effects, LIPUS also induces both cyclic and non-cyclic alterations in living tissues [4]. One of the primary non-cyclic effects attributed to LIPUS therapy is acoustic microstreaming, a phenomenon that perturbs the local cellular environment by altering concentration gradients in the vicinity of the cell membrane [11, 21–23]. Such changes are believed to impact ion diffusion across the cell membrane, thereby promoting fluid flow and circulation. This, in turn, facilitates the redistribution of essential nutrients, oxygen, and signaling molecules [24].

A growing body of in vitro and in vivo research has illuminated the complex biological pathways activated by LIPUS in living tissues. For instance, experimental evidence reveals that ultrasound stimulation modulates the flux rates of potassium ions within rat thymocytes, providing insight into its effects on ion transport mechanisms [11]. Moreover, LIPUS has been implicated in the acceleration of fracture healing, a process mediated, at least in part, by intracellular calcium signaling pathways [25]. Other studies have identified a regulatory role for ultrasound therapy in the production of key signaling molecules, including transforming growth factor β (TGF-β), interleukin 6 (IL-6), and tumor necrosis factor α (TNF-α). Specifically, ultrasound-induced upregulation of TGF-β by osteoblasts, coupled with concomitant reductions in IL-6 and TNF-α concentrations, contributes to the prevention of bone loss [26]. Furthermore, the ability of ultrasound to stimulate the release of vascular endothelial growth factor (VEGF) underscores its potential impact on endothelial cell proliferation and migration [27, 28].

**Fig. 1:**
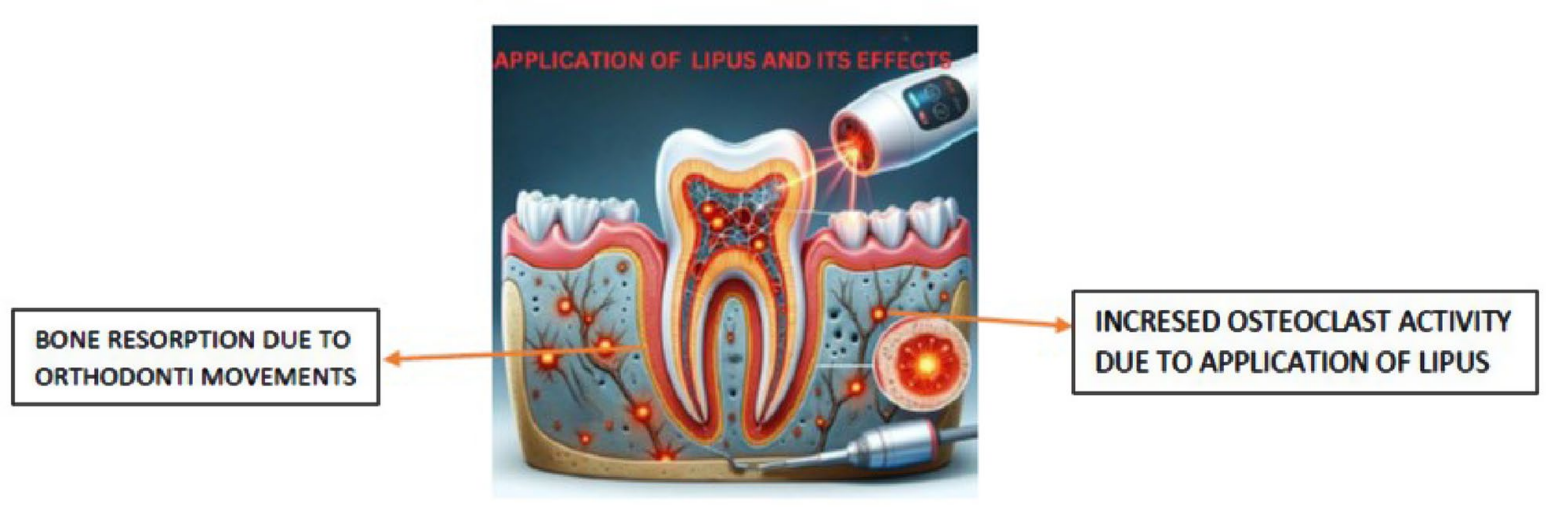
Mechanism of root resorption

Root resorption is a significant concern in dentistry, particularly in orthodontic treatments. It involves the loss of dental root structure, primarily due to prolonged mechanical stress or injury. Low-Level Therapy (LLT), particularly Low-Intensity Pulsed Ultrasound (LIPUS), has emerged as a promising approach in addressing this issue. [12] LLT, through its non-invasive ultrasound waves, is believed to modulate cellular activities in the periodontal ligament and surrounding bone tissue. This modulation aids in mitigating the inflammatory response and enhancing cellular repair processes, which are crucial in preventing or limiting root resorption. The use of LIPUS in this context not only helps in preserving the integrity of the dental roots during orthodontic treatments but also promotes quicker recovery and regeneration of the affected tissues. This application of LIPUS showcases its potential as a versatile tool in dental therapy, offering a means to protect and regenerate vital dental structures, thereby enhancing the overall outcomes of various dental treatments. [12, 29, 30] (Fig. 1).

This systematic review aims to comprehensively analyze and synthesize existing scientific evidence on the efficacy of low-intensity pulsed ultrasound therapy in the treatment or prevention of orthodontically induced inflammatory root resorption (OIIRR). By providing a rigorous evaluation of the existing literature, we seek to understand the underlying mechanisms, applications, and effectiveness of this therapeutic intervention in a clinical orthodontic context.

### Objectives

1. Search and collect all available studies, both randomized and non-randomized, investigating the use of low-intensity pulsed ultrasound therapy for OIIRR, from various electronic databases and other sources.
2. To evaluate the methodological quality, study design, and risk of bias in the selected studies, using recognized tools and criteria.
3. To formulate evidence-based recommendations for the application of low-intensity pulsed ultrasound therapy in clinical orthodontic practice, considering its effectiveness and safety profile in the context of OIIRR.

## Materials and methods

### Protocol and ethics

**Fig. 2:**
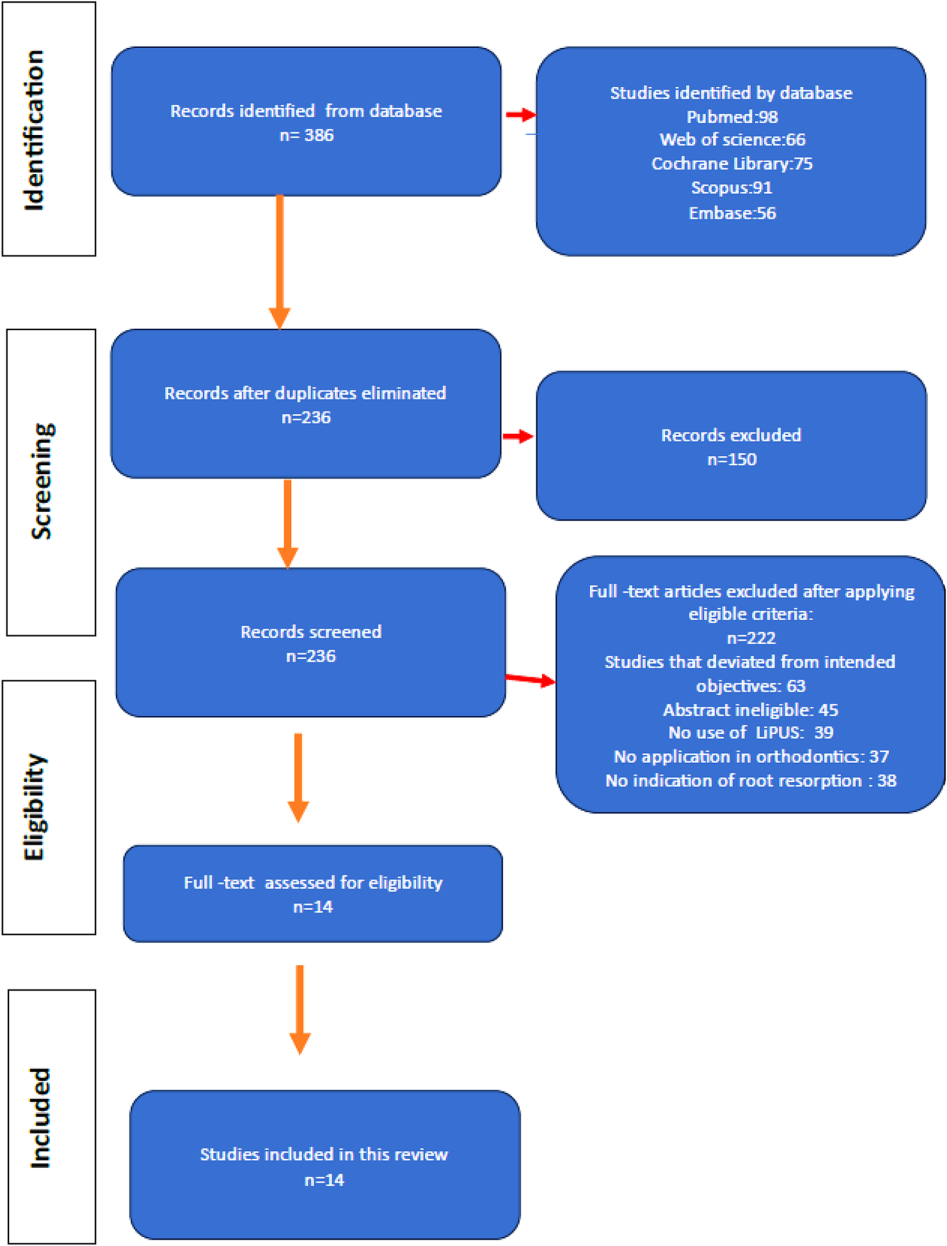
PRISMA guideline time table and process

The Cochrane technique was used to conduct this systematic review protocol, following the Preferred Reporting Items for Systematic Reviews and Meta-Analyses (PRISMA) reporting standards (Fig. 2).

The PICO strategy was implemented to define the type of papers to be selected for the review.

- **P (Population)**: Individuals undergoing orthodontic treatment who are at risk of or are experiencing orthodontically induced inflammatory root resorption (OIIRR). This could include patients across various age groups, genders, and backgrounds, with different types and stages of orthodontic treatment.
- **I (Intervention)**: The application of low-intensity pulsed ultrasound therapy as a treatment or preventive measure for OIIRR. The parameters of the therapy such as intensity, frequency, duration, and timing relative to orthodontic treatment can be examined.
- **C (Comparison)**: Other conventional or experimental treatments for OIIRR, or the absence of such treatments (e.g., standard orthodontic care without additional interventions for OIIRR). This can help to determine the relative efficacy of low-intensity pulsed ultrasound therapy.
- **O (Outcome)**: The primary outcomes might include the prevention or reduction of OIIRR, improvement in dental health, or reduction in treatment complications. Secondary outcomes could include patient satisfaction, side effects, or cost-effectiveness.

This review was registered under the following provisional PROSPERO ID number: CRD42023445179.

### Search protocol

A comprehensive automated literature search was performed in major databases including MEDLINE(via PubMed), EMBASE, PsycINFO, Web of Knowledge, Scopus, CINAHL, LILACS, SciELO, Cochrane, trials registries,3ie, and Google Scholar covering the period from January 2009 to April 2023. The search strategy involved the use of specific keywords and Boolean operators. The following combinations were employed: “LIPUS” and “Orthodontically induced root resorption,” “LIPUS” and “root resorption” and “hard-tissue healing,” “low-intensity pulsed ultrasound” and “orthodontic treatment,” “low-intensity pulsed ultrasound” and “Orthodontically induced root resorption,” “low-intensity pulsed ultrasound” and “hard tissue” and “Orthodontically induced root resorption.“(Table 1) In addition to the electronic searches, manual screening of the reference sections of identified articles was conducted to identify any additional relevant publications.

**Table 1:** Search criteria and terminology used for various databases

S.NO	DATABASE	SEARCH TERMINOLOGY
1	MEDLINE(Via PubMed)	((“ULTRASONIC WAVES“[Title/Abstract] OR therapeutic ultrasound“[Title/Abstract] OR " ULTRASONIC WAVES “[MeSH Terms] OR “ULTRASONIC WAVES “[MeSH Terms]) AND “root resorption“[Title/Abstract] OR “hard-tissue healing“[Title/Abstract] OR “orthodontic treatment“[Title/Abstract] OR “resorption“[MeSH Terms] OR “root resorption“[MeSH Terms] OR “hard-tissue healing“[MeSH Terms] OR “orthodontic treatment“[MeSH Terms])) OR ((“root resorption“[All Fields] OR “root resorption“[MeSH Terms]) ((“orthodontics“[MeSH Terms] OR “tooth movement“[All Fields]) OR (“orthodontics“[MeSH Terms] OR “orthodontics“[All Fields])))
2	EMBASE	1. “root resorption”.mp. and exp tooth disease/ 2. exp orthodontics/ 3. #1 and #2 4. “tooth movement”.mp. 5. #2 or #4 6. #1 and #5 7. ‘hard-tissue healing’:ti,ab,kw OR ‘orthodontic treatment’:ti,ab,kw))
3	PsycINFO	((“ Ultrasound“[Mesh] OR “ultrasound therapy” OR “therapeutic ultrasound”) AND (“Root Resorption“[Mesh] OR “orthodontics”OR “root resorption” OR “Tooth Movement”) AND (“Systematic Review“[Publication Type] OR “systematic review”))
5	Scopus	TITLE-ABS-KEY(“ultrasound therapy” AND “orthodontics” AND “therapeutic ultrasound” AND “root resorption” AND “Tooth Movement” AND “systematic review”)
6	CINAHL	MH (“therapeutic ultrasound”)AND MH (“orthodontics”) AND MH (“root resorption”) AND MH (“systematic review”) AND MH (“Tooth Movement”)
7	SciELO	(“ ultrasound " AND “orthodontics” AND “ root resorption” AND “systematic review” AND “therapeutic ultrasound” AND “Tooth Movement”)
8	Cochrane	1. MeSH descriptor: [Root Resorption] this term only 2. MeSH descriptor: [Orthodontics] 1 tree(s) exploded 3. “root resorption” 4. orthodontics 5. “tooth movement” 6. #1 or #3 7. #2 or #4 or #5 8. #6 and #7
9	LILACS	(tw:(root resorption OR Resorción Radicular OR Reabsorção da Raiz OR Reabsorción de Raíces Dentales OR Reabsorción Radicular OR Resorción de la Raíz Dental OR Resorción de Raíz Dental)) AND (tw:(orthodontics OR ortodoncia OR orthodontia)) OR (tw:(tooth movement OR Movimiento dentario OR Movimiento de los Dientes OR Movimiento de un Diente OR Movimiento Ortodóncico OR Pequeños Desplazamientos de los Dientes OR Movimiento Dentario Menor OR Levantamiento Dentario OR Movimentação Dentária OR Verticalização Dentária OR Movimento dos Dentes OR Movimento de um Dente OR Movimentação de Dentes OR Movimentação Ortodôntica OR Movimento Ortodôntico)) AND NOT (tw:(endodontics)) AND NOT (tw:(calcium hydroxide)) AND NOT (tw:(replantation)) AND NOT (tw:(autotransplantation))

### Exclusive criteria

Studies that utilized an ultrasound intensity of 0.1 W/cm² were excluded from the review, along with non-subject-related English publications. Studies that deviated from intended objectives and studies categorized as case-control (evidence level 3) and case-series (evidence level 4) where the primary action of LIPUS was not aiming to treat OIIRR were not included in the evaluation.

### Inclusion criteria

The focus of the search was on systematic reviews and meta-analyses of randomized controlled trials (evidence level 1a), randomized controlled trials (evidence level 1b), clinical trials without randomization (evidence level 2a), and other experimental studies (evidence level 2b).

### Data extraction protocol and quality of evidence

The data collection and extraction process commenced with a meticulous screening of the titles and abstracts of the identified studies, conducted independently by the authors to ensure consistency and concordance. Articles deemed potentially relevant, based on their titles and abstracts, were further subject to an exhaustive and independent manual assessment, involving the full-text review.

The following specific data were meticulously extracted from each study: the publication year, country of origin, detailed description of the sample including size, age, gender, and habit profile, comprehensive study design encompassing type and intervention modalities, precisely measured outcomes, follow-up information, and a thorough examination of the statistical analyses employed.

This rigorous approach ensures a robust and transparent methodology, aligned with the stringent standards required for a high-level scientific investigation, contributing to the objective synthesis of the existing evidence on low-intensity pulsed ultrasound therapy in orthodontically induced inflammatory root resorption.

**Fig. 3:**
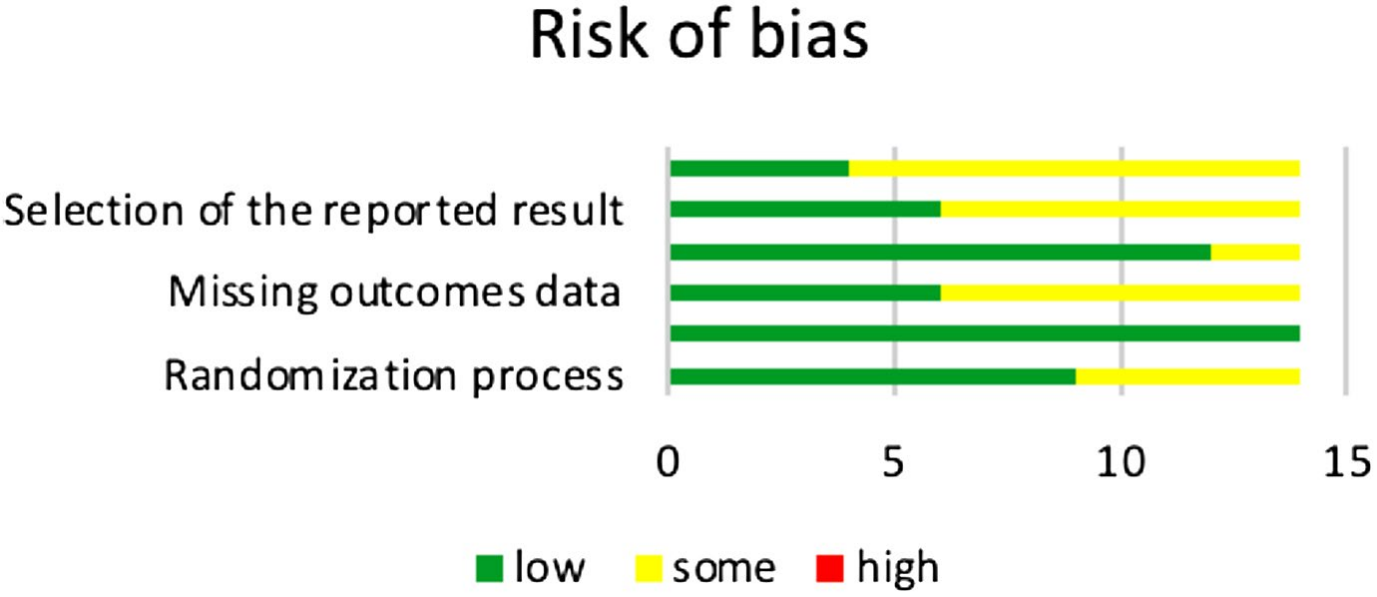
Risk of bias analysis

The overall quality of evidence of RCTs for each outcome was assessed and reported using the GRADE approach. After data extraction was completed, we assessed the risk of bias within each study. The assessment was carried out independently by the same two reviewers using the revised Cochrane risk of bias tool (RoB 2.0) and we resolved disagreements regarding the risk of bias through discussions. (Tables 2 and 3, and Fig. 3).

**Table 2:** Risk of bias analysis

	Low	Medium	High
Randomization process	9	5	0
Deviations from intended interventions	14	0	0
Missing outcomes data	6	8	0
Measurement of the outcomes	12	2	0
Selection of the reported result	6	8	0
Overall	4	10	0

**Table 3:** Risk of bias analysis based on all the studies analyzed

Sl. No	Study	Randomization Process	Deviations from intended interventions	Missing outcomes data	Measurement of the outcomes	Selection of the reported result	Overall
1	El-bailey et al.	+	+	+	+	+	+
2	Bona et al.	+	+	+	+	?	+
3	Inbushi et al.	+	+	+	+	+	+
4	Scheven et al.	+	+	?	+	?	+
5	Rego et al.	+	+	+	+	+	+
6	El-bailey et al. clinical trial	?	+	?	+	?	+
7	Al-Dagheer et al.	?	+	+	+	?	+
8	Liu et al.	+	+	?	+	+	+
9	Wierbicki et al.	+	+	?	?	?	?
10	Vafaeian et al.	+	+	+	+	+	+
11	Chan et al.	?	+	?	+	?	?
12	Xue et al.	+	+	?	+	+	+
13	Barley et al.	?	+	?	?	?	?
14	Casa et al.	?	+	?	+	?	?

Low risk: + Some concerns: ? High risk: -

## Results

Within the confines of the selection criteria, a total of 14 studies were deemed suitable for inclusion in this systematic review. The corpus of studies encompasses a diverse array of research designs, with three being randomized controlled trials, while the remaining 11 constitute either clinical trials or controlled clinical trials.

The investigations centered on the effects of Low-Intensity Pulsed Ultrasound (LIPUS) therapy on orthodontically induced root resorption (RR) and the subsequent reparative processes. A nuanced analysis of the selected studies yielded several notable observations:

- Six of the studies presented evidence indicating that LIPUS therapy may serve as an efficacious medical intervention, demonstrating potential in both shielding against root resorption and facilitating its repair.
- A subset of four studies directed their inquiry toward the stimulatory effects of LIPUS therapy on cementoblasts, unveiling its potential role in enhancing their proliferation or activity.
- A further triad of studies reported a quantifiable decrease in the instances of RR within the cohorts subjected to LIPUS treatment.

These findings collectively provide a multifaceted insight into the applications and efficacy of LIPUS therapy in the context of orthodontically induced root resorption, delineating its potential therapeutic benefits, and laying a foundation for further exploration in this domain.

A summary of the results from all the included studies can be found in Table 4.

**Table 4:** Summery of all the included studies

Sl no	Reference	Author & year	LIPUS dosage	Result(s)
1	48	El-Bialy et al.		LIPUS combined with distraction enhanced mandibular incisor growth and eruption.
2	50,53	Bona et al.	150 mW/cm(2).	Single ultrasound exposure increased the expression of transcripts for COL-I and ALP after 24 h; the expression of OPG and RANKL also increased after 1 and 4 h, respectively. Cultured cementoblasts receiving ultrasound stimulation for 6 days showed a significant (p < 0.05 or 0.01) increase in cell number and collagen synthesis. ALP activity and OPG synthesis were also significantly (p < 0.05) upregulated by ultrasound stimulation with 150 mW/cm(2). These results demonstrated that ultrasound stimulation especially with 150 mW/cm(2) might be a better candidate as a medical remedy to protect against root resorption and/or accelerate its repair.
3	25,34	Inubushi et al.	30 mW/cm2	LIPUS exposure significantly reduces root resorption by the suppression of cementoclastogenesis by altering OPG/RANKL ratio during orthodontic tooth movement without interfering tooth movement. LIPUS may be an effective tool to prevent root resorption during tooth movement and is applicable to clinical use in near future.
4	54	Scheven et al.	30 mW/cm2	The effect of ultrasound on odontoblast-like cells and observed that the expression of Collagen-I, Osteopontin (OPN), TFGβ1 and heat shock protein (hsp) increased after ultrasound application. An interesting finding of this study was the effect of ultrasound on hsp 25/27, suggesting the role of this protein in response of odontoblasts to ultrasound
5	35,56	Rego et al.	30 mW/cm2	Increased PGE2 formation in LIPUS (30 mW/cm2) treated cells compared to control. Also, the gene expression levels of ALP, bone morphogenic protein 2 (BMP-2) and osteopontin (OPN) significantly increased in LIPUS treated group compared to control. In addition, LIPUS stimulation upregulated the mRNA expression levels of EP2 and EP4, however, the mRNA expression levels of EP1 and EP3 which were unaffected, thus, suggesting that LIPUS therapy can promote cementoblast differentiation through EP2/EP4 receptor pathway(
6	31	El-Bialy et al.- clinical trial	30 mW/cm2	The stimulatory effect of LIPUS on cementum regeneration and repair. Using a split mouth design, they tipped the premolars facially with an initial force level of 50 gm accompanied with LIPUS therapy (30 mW/cm2) on left side whereas the right side was used as a control. LIPUS was applied for 20 min/day for four weeks after which the teeth were extracted and were analyzed. A significant decrease in the number and surface area of resorption lacunae was observed in the LIPUS treated premolars compared to control. Also LIPUS treated premolars showed significant deposition of new cellular cementum (reparative cementum) compared to control
7	32	Al-Daghreer et al.	30 mW/cm2	Observed less root resorption in the LIPUS (30 mW/cm2) treated teeth compared to control. They reported that number of resorption lacunae, total volume of resorption lacunae and percentage of tooth root resorption was significantly less in the LIPUS group compared to control. Also, they observed deposits of new cellular cementum on LIPUS treated roots compared to control. Moreover, they also noted that LIPUS treated group exhibited significantly thicker cementum on middle and apical third of the root compared to control. The distribution of osteoclast and odontoclast along the root surfaces were also reported.
8	12	Liu et al.		Rat model also reported decreased number of RL in the LIPUS treated teeth compared to contro
9	52	Wierzbicki et al.		The mean percentage of root resorption of the teeth after undergoing one year of regular orthodontic treatment to be 0.88% compared to 0.55% of the control group in this study, where the teeth were subjected to a fairly low level of torque for only 4 weeks. This further signifies the deleterious effect of torque on root resorption.
10	57	Vafaeian et al.		The quantitative relationship between the thicknesses of regenerated cementum and ultrasound power. He reported a non-uniform distribution of ultrasound pressure amplitudes on different root surfaces.
11	61	Chan et al. clinical study		Root resorption with heavy tensile forces
12	62	Xue et al.		Rat model demonstrated that LIPUS can accelerate orthodontic tooth movement via activation of Bone Morphogenic Protein-2 (BMP-2) signalling pathway
13	63	Barley et al.		Applied 2.85 N-mm (285 g-mm) of torque and observed more resorption at the apical level than at middle and cervical level.
14	64	Casa et al.		Applied 6 N-mm of torque and reported severe root resorption at the apex.

## Discussion

The therapeutic effects of Low-Intensity Pulsed Ultrasound (LIPUS) application on bone remodeling have been substantiated through extensive research. In vivo studies present a compelling body of evidence illustrating the capacity of LIPUS to enhance bone regeneration and repair [31], expedite the healing process of bone fractures, and stimulate osteogenesis at distraction sites [32]. These findings have culminated in the clinical establishment of LIPUS stimulation, resulting in its wide application within medical practice. Moreover, the United States Food and Drug Administration (FDA) has bestowed formal approval upon LIPUS as a method for accelerating fracture healing [5]. This recognition underlines the significant therapeutic potential of LIPUS, underscoring its role as an innovative and effective treatment modality in the field of orthopedics and beyond.

In the realm of dentistry, the utilization of ultrasound therapy represents a relatively nascent development in comparison to its established presence in the broader field of medicine. With a history extending back approximately a decade, ultrasound therapy in dental research has begun to manifest its potential, demonstrating stimulating effects across an array of cellular types. These include cementoblasts [33], odontoblast-like cells [34], osteoblasts [31], chondrocytes [35], gingival cells [36, 37], and periodontal ligament [30].

Low-Intensity Pulsed Ultrasound (LIPUS) has been revealed as particularly effective in circumventing root resorption attributable to orthodontic tooth movement [12, 38–40], as well as tooth re-implantation [41]. Beyond these applications, LIPUS has contributed to improved outcomes in bone healing during human sinus augmentation procedures [42] and animal mandibular distractions [43, 44].

The stimulating influence of LIPUS on periodontal ligament cells has garnered scholarly attention. One illustrative in vitro study by Inubushi et al. [33] shed light on the induction of a marked differentiation of immature cementoblasts and a concomitant increase in alkaline phosphatase (ALP) activity when immature cementoblast cells were exposed to LIPUS. These findings herald potential avenues for periodontal tissue regeneration and repair [33]. Further investigation has highlighted the capacity of LIPUS to foster periodontal tissue regeneration post-injury [45] and surgery [46], as well as its stimulatory effects on odontoblast cells [34].

LIPUS’s dynamic therapeutic potential has been further underscored by studies indicating its role in stimulating odontoblast cells to secrete pre-dentin [29, 47–49]. In a particularly intriguing development, a recent exploration by El-Bialy et al. [50] has unveiled the possibility of differentiating gingival multipotent cells into neural cells through LIPUS. This finding might have significant implications, extending the horizon of dental pulp tissue engineering techniques and reinforcing the versatile and transformative role of LIPUS within dental medicine.

### Ultrasound and orthodontically induced inflammatory root resorption (OIIRR)

Cementum, a specialized mineralized tissue, enshrouds the outer surface of the tooth’s root, acting as a pivotal anchor linking the teeth to the adjacent alveolar bone. While bearing a compositional resemblance to bone, cementum exhibits distinct structural and functional attributes [33]. One striking difference lies in its limited potential for remodeling [51], a characteristic that can be further constrained by pathological conditions such as disease or inflammation [52–54].

Given these challenges, recent years have witnessed an escalating interest among clinicians in exploring the potential for cementum regeneration through ultrasound. El-Bialy et al. [55] illuminated this pathway through an experimental rabbit model, demonstrating that Low-Intensity Pulsed Ultrasound (LIPUS) application enhances mandibular growth, facilitating root formation and continuous incisor eruption.

At the cellular level, cementoclastogenesis, or the process of cementum resorption, is governed by a delicately balanced interplay between receptor activator of nuclear factor-kappa B ligand (RANKL) and osteoprotegerin (OPG) within cementoblasts [56]. This equilibrium determines cementoclast activity, where an elevated RANKL/OPG ratio fuels cementoclastogenesis, while a decrease exerts the opposite effect.

Building upon this understanding, an insightful in vitro study by Bona et al. [57] uncovered that ultrasound not only inhibits orthodontically induced inflammatory root resorption (OIIRR) by dampening cementoclastogenesis but also fosters cementum regeneration and repair. Intriguingly, they observed a reduced RANKL/OPG ratio in ultrasound-treated cells (150 mW/cm²) relative to the control, culminating in a diminution of cementoclast activity and a subsequent alleviation of OIIRR. Further, a proliferation of cementoblast cells was noted upon exposure to ultrasound (30 mW/cm² and 150 mW/cm²) compared to the control group [57].

This nuanced understanding of ultrasound’s dual role in both inhibiting resorption and promoting regeneration illuminates a promising avenue for therapeutic intervention, offering a sophisticated tool in the evolving landscape of dental treatment modalities.

Inubushi et al. [40] contributed analogous and insightful findings to this burgeoning field of study. Through an in vitro experiment, they delineated the effects of Low-Intensity Pulsed Ultrasound (LIPUS) exposure (30 mW/cm²), revealing an increase in RANKL mRNA expression levels in both cementoblasts and osteoblasts. Strikingly, OPG mRNA expression levels were exclusively elevated in cementoblasts, hinting at LIPUS’s role in encouraging osteoclastogenesis while simultaneously averting cementoclastogenesis [40].

Furthermore, after two weeks of experimental force application, the LIPUS group (150 mW/cm²) exhibited a substantial reduction in the number of odontoclast cells and a corresponding increase in osteoclast cells relative to the control group. Concomitant with this finding was a discernable decrease in the resorption area and an enhancement of root thickness within the LIPUS group (150 mW/cm²), compared to controls [40]. The augmentation of osteoclastic activity under the influence of LIPUS was also substantiated by El-Bialy et al. [48].

In another intriguing in vitro assessment, the LIPUS group (30 mW/cm²) displayed elevated osteoclasts within the periodontal ligament compared to controls, intimating that LIPUS therapy might be an instrumental adjunct in expediting orthodontic tooth movement. Accompanying this was an observed thickening of both cementum and predentin in LIPUS-treated cells relative to the control group [48].

Beyond these specific findings, the underlying molecular mechanisms warrant attention. Alkaline phosphatase (ALP), ubiquitously present across body tissues, is instrumental in mineralized tissue calcification and serves as an early phenotype marker for mature cementoblasts [57]. Likewise, Type I collagen (COL-I), a fundamental constituent of bone and the extracellular matrix (ECM), has its synthesis tightly intertwined with the genesis of differentiated and mineralized tissues [58]. Moreover, Runx-2, an osteoblast-specific transcription factor pivotal in regulating osteoblast differentiation and gene expression [59], is conjectured to exert parallel influences on cementoblast differentiation, akin to its effects on osteoblasts [33].

In a sophisticated in vitro study undertaken by Inubushi et al. [33], a significant elevation in the expression levels of Alkaline Phosphatase (ALP), Type I Collagen (COL-I), and Runx-2 mRNA was discerned in cells treated with Low-Intensity Pulsed Ultrasound (LIPUS) at 30 mW/cm² relative to the control group. This investigation further revealed a marked upswing in ALP activity, Runx-2 protein levels, and collagen synthesis concomitant with LIPUS exposure, elucidating its potential to foster the repair and regeneration of cementum.

Complementing these findings, Bona et al. [60] detected an enhancement in the expression levels of ALP mRNA (150 mW/cm²) and a surge in calcium content (100 mW/cm² and 150 mW/cm²) in ultrasound-exposed cells compared to controls. Scheven et al. [61] further extended this body of knowledge through a concise in vitro examination of ultrasound’s impact on odontoblast-like cells. They unveiled a pronounced increase in the expression of Collagen-I, Osteopontin (OPN), TGF-β1, and heat shock protein (hsp) following ultrasound application, with the role of heat shock proteins 25/27 in odontoblast response to ultrasound emerging as a novel and intriguing observation [61].

Beyond the cellular level, prostaglandin E2 (PGE2) represents a vital signaling molecule, renowned for its governance over bone metabolism. PGE2 exerts its effects via activation of specific receptors, including EP1, EP2, EP3, and EP4 [62], with the latter two playing a cardinal role in orchestrating bone formation [55]. In an innovative in vitro exploration, Rego et al. [63] identified an amplified formation of PGE2 in LIPUS-treated cells (30 mW/cm²) compared to controls. Remarkably, they also ascertained a substantial increase in the gene expression levels of ALP, bone morphogenetic protein 2 (BMP-2), and osteopontin (OPN) in the LIPUS cohort relative to controls [63]. Further shedding light on the underlying molecular pathways, LIPUS stimulation was found to selectively augment the mRNA expression levels of EP2 and EP4 receptors, leaving EP1 and EP3 receptors undisturbed. This indicates that LIPUS therapy may be adept at propelling cementoblast differentiation via the specific modulation of the EP2/EP4 receptor pathway [63].

Beyond the confines of in vitro experimentation, in vivo investigations have been conducted to scrutinize the impact of Low-Intensity Pulsed Ultrasound (LIPUS) on orthodontically induced inflammatory root resorption (OIIRR). In a rigorously designed clinical trial led by El-Bialy et al. [38], the regenerative capacity of LIPUS in enhancing cementum repair was elucidated. This study embraced a split-mouth design, wherein the left side premolars were subjected to an initial orthodontic force of 50 g, synergistically combined with daily LIPUS therapy at 30 mW/cm² for 20 min over four weeks. The right side of the mouth served as a carefully matched control.

Following this intervention, teeth were meticulously extracted and submitted for comprehensive analysis. Remarkably, the LIPUS-treated premolars exhibited a pronounced reduction in both the number and surface area of resorption lacunae compared to their control counterparts. In a compelling affirmation of LIPUS’s therapeutic potential, the treated premolars were also marked by a significant accretion of new cellular cementum, termed reparative cementum, a finding that was conspicuously absent in the control group [64–67].

The multifaceted role of Low-Intensity Pulsed Ultrasound (LIPUS) in mitigating root resorption has been rigorously interrogated through both in vitro and in vivo studies. In an insightful experimental study employing a canine model, Al-Daghreer et al. [39] assiduously investigated the potential of LIPUS-treated teeth (30 mW/cm²) to counter root resorption. Their results were marked by a significantly lower incidence of resorption lacunae, diminished total volume of these lacunae, and a reduced overall percentage of tooth root resorption in the LIPUS group as juxtaposed with the control group. Moreover, deposition of new cellular cementum was detected on the LIPUS-treated roots, accompanied by thicker cementum formation in both the apical and middle thirds of the root compared to the control. Fascinatingly, the distribution of osteoclasts and odontoclasts along the root surfaces unveiled a significantly higher prevalence of osteoclast cells in the middle and apical thirds of the LIPUS-treated root, while odontoclast cells were more pronounced in the control group [39]. These observations resonate with prior elucidations concerning the dichotomous effects of LIPUS on osteoclastogenesis and cementoclastogenesis [40, 48].

Echoing these findings, Liu et al. [12] pursued an experimental approach in a rat model, extending our comprehension of the role of LIPUS in dental care. With meticulous scrutiny, they discerned that the total count and surface area of resorption lacunae were markedly elevated in the positive control group (subjected solely to orthodontic tooth movement without LIPUS intervention) relative to the ultrasound groups (at intensities of 100 and 150 mW/cm²).

In the realm of orthodontic research, the innovative application of Low-Intensity Pulsed Ultrasound (LIPUS) has provided intriguing insights into cementum regeneration. Liu et al. [12] embarked on a comprehensive investigation, employing LIPUS intensities of 100 and 150 mW/cm², to discern the effects on new cementum formation. Their findings elucidated that both LIPUS-treated groups manifested substantial new cementum formation, with a concomitant and significant reduction in the RANKL/OPG ratio in the 100 mW/cm² ultrasound group, when contrasted with the positive control group.

Complementing these insights, Wierzbicki et al. [59] meticulously assessed the ramifications of one year of regular orthodontic treatment, contrasting a mean percentage of root resorption of 0.88% against a mere 0.55% in the control group. Intriguingly, their study design encompassed the application of relatively low-level torque over a mere four-week duration, thereby accentuating the insidious and deleterious impact of torque on root resorption.

While the resorption process has been traditionally quantified by enumerating the resorption lacunae on each root surface, recent investigations have illuminated that the mere number of lacunae does not conclusively delineate the severity of the process [68–71]. However, in the LIPUS-treated groups, the reduction in the number of resorption lacunae across all root surfaces, when juxtaposed with the control group, serves as a testament to its potential efficacy. The underlying anti-inflammatory properties of LIPUS may indeed be instrumental in mediating this effect.

In the continuing exploration of Low-Intensity Pulsed Ultrasound (LIPUS) in dental applications, Vafaeian et al. [68] made significant strides through finite element model analysis. Their work illuminates the complex quantitative relationship between the thickness of regenerated cementum and ultrasound power, revealing a nuanced, non-uniform distribution of ultrasound pressure amplitudes on varying root surfaces. This phenomenon offers compelling insights into the disparate stimulatory and inhibitory effects of LIPUS across different root surfaces, where higher ultrasound pressure culminates in increased cementum thickness, and conversely, reduced exposure leads to diminished thickness, especially on the surfaces nearest and farthest from the ultrasound transducer [72–77].

Complementing these findings, Chan et al. [78] uncovered the consequences of heavy tensile forces, leading to root resorption. Meanwhile, in an experimental rat model, Xue et al. [79] furnished compelling evidence that LIPUS can expedite orthodontic tooth movement, implicating the activation of the Bone Morphogenetic Protein-2 (BMP-2) signaling pathway as the underlying mechanism.

Adding to the complexity of this field, Barley et al. [80] applied a torque of 2.85 N-mm, observing a predilection for resorption at the apical level, as contrasted with the middle and cervical levels. Similarly, Casa et al. [81] applied a torque of 6 N-mm, and their observations painted a stark picture of severe root resorption at the apex.

The application of Low-Intensity Pulsed Ultrasound (LIPUS) has been shown to markedly reduce the severity of orthodontically induced inflammatory root resorption (OIIRR) through the enhancement of cementum repair. However, it’s imperative to recognize that LIPUS may not fully heal the resorption craters within the standard treatment period. Therefore, considerations for extending the time interval between LIPUS activations are particularly warranted, especially for patients at elevated risk for OIIRR or those exhibiting root resorption during the initial stages of orthodontic intervention. This tailored approach aims to facilitate the healing of resorbed cementum and thwart further root resorption.

Long-term clinical trials rigorously evaluating the impact of LIPUS on OIIRR would significantly contribute to our understanding of the therapy’s efficacy in expediting cementum regeneration and repair over an extended duration. Such research has the potential to further refine treatment protocols, aligning them more precisely with individual patient needs.

Future long-term randomized clinical trials are necessitated to explore the effects of Low-Intensity Pulsed Ultrasound (LIPUS) on orthodontically induced inflammatory root resorption (OIIRR) with a specificity that reflects the time frame of regular orthodontic treatment duration, often spanning two years. These nuanced investigations promise to shed further light on the multifaceted dynamics of LIPUS, including its stimulatory and inhibitory impacts on cementogenesis and cementoclastogenesis.

Moreover, the studies hold the potential to deepen our comprehension of the interplay between orthodontic force and OIIRR, allowing for a more intricate understanding of these complex biological processes. Crucially, they will offer an opportunity to assess the tangible damage sustained by teeth subjected to standard orthodontic treatment, providing a more robust and comprehensive foundation for evaluating and improving clinical practices.

The realization of these long-term trials would mark a significant advancement in the field, potentially guiding clinical strategies and enhancing the precision and efficacy of LIPUS in mitigating the challenges associated with OIIRR. By rigorously probing the subtleties of these phenomena, clinicians and researchers can work collaboratively toward more personalized and effective therapeutic interventions, ensuring alignment with the highest standards of patient care and scientific inquiry.

## Conclusion

Orthodontic treatment, while critical in the correction of malocclusions, may entail undesirable complications such as Orthodontically Induced Inflammatory Root Resorption (OIIRR), a condition that leads to the irreversible loss of root structure. The challenge of mitigating this deleterious effect necessitates the exploration of non-invasive adjuvant modalities that can be harnessed during the torque application phase of orthodontic treatment.

Low-Intensity Pulsed Ultrasound (LIPUS) has emerged as a promising intervention in this regard, displaying clinically meaningful results in reducing the severity of OIIRR. The underlying mechanisms of this therapeutic approach encompass a rich interplay of biological processes that act to repair and regenerate the affected root tissues.

Investigations evaluating the efficacy of LIPUS in the context of OIIRR have not only expanded our understanding of the intricate relationship between orthodontic forces and root resorption but have also illuminated the potential of LIPUS as both a preventive measure and treatment option. These insights hold substantial value for the clinical community, offering a refined perspective on the nature of the damage incurred by teeth during conventional orthodontic procedures.

Future Perspectives of the Study: The realization of long-term trials will significantly advance LIPUS applications in addressing OIIRR. Future research should focus on optimizing LIPUS treatment parameters, combining it with other therapies, and examining its long-term dental impacts. These efforts are poised to transform orthodontic care, particularly for patients susceptible to OIIRR, enhancing treatment efficacy and patient outcomes.

## Data Availability

The data will be available on reasonable request from the corresponding author.

## Abbreviations

RF: Radiofrequency
LIPUS: Low-intensity pulsed ultrasound
PEMF: Pulsed electromagnetic field
OIIRR: Orthodontically induced inflammatory root resorption
RR: Root resorption
RCT: Randomized controlled trial
FAK: Focal adhesion kinase
MAPK: Mitogen-activated protein kinase
COX-2: Cyclooxygenase-2
MAPK: Mitogen-activated protein kinase
TGF-β: Transforming growth factor β
IL-6: Interleukin 6
GRADE: Grading of Recommendations Assessment, Development, and Evaluation
TNF- α: Tumor necrosis factor α
VEGF: Vascular endothelial growth factor
FDA: Food and Drug Administration
ALP: Alkaline phosphatase
RANKL: Receptor activator of nuclear factor-kappa B ligand
OPG: Osteoprotegerin
ECM: Extracellular matrix
COL-1: Type I collagen
RUNX-2: Osteopontin
PGE 2: Prostaglandin E2
EP: Eicosapentaenoic acid
BMP-2: Bone morphogenetic protein 2
RNA: Ribonucleic acid
